# Prediction and Chemical Interpretation of Singlet-Oxygen-Scavenging Activity of Small Molecule Compounds by Using Machine Learning

**DOI:** 10.3390/antiox10111751

**Published:** 2021-11-01

**Authors:** Taiki Fujimoto, Hiroaki Gotoh

**Affiliations:** Department of Chemistry and Life Science, Yokohama National University, 79-5 Tokiwadai, Hodogaya-ku, Yokohama 240-8501, Japan; fujimoto-taiki-gc@ynu.jp

**Keywords:** machine learning, antioxidant, singlet oxygen, feature importance, interpretability, carotenoid

## Abstract

A chemically explainable machine learning model was constructed with a small dataset to quantitatively predict the singlet-oxygen-scavenging ability. In this model, ensemble learning based on decision trees resulted in high accuracy. For explanatory variables, molecular descriptors by computational chemistry and Morgan fingerprints were used for achieving high accuracy and simple prediction. The singlet-oxygen-scavenging mechanism was explained by the feature importance obtained from machine learning outputs. The results are consistent with conventional chemical knowledge. The use of machine learning and reduction in the number of measurements for screening high-antioxidant-capacity compounds can considerably improve prediction accuracy and efficiency.

## 1. Introduction

Oxygen is essential for human life. However, in the human body, some amount of oxygen exists as reactive oxygen species, which contributes to the immunity mechanism. However, aging and carcinogenesis are also attributed to reactive oxygen species because of their high reactivity [1]. Antioxidants are substances that remove reactive oxygen species in the body, and the ability to scavenge reactive oxygen species is called antioxidant capacity. Therefore, dietary intake of antioxidants through vegetables, fruits, crustaceans, and other foods is recommended [2].

The method for measuring the scavenging activity depends on the target reactive oxygen species. Furthermore, the use of only one method for determining the antioxidant capacity of compounds and foods may not provide reliable results. The oxygen radical absorbance capacity (ORAC) assay [3,4] was developed as an antioxidant capacity assay for peroxyl radicals, which are commonly used as an indicator of the antioxidant capacity of foods and compounds [5]. Phenolic antioxidants exhibit high radical-scavenging capacity. However, carotenoids exhibit low peroxyl radical-scavenging ability and high singlet-oxygen-scavenging ability [6]. The singlet-oxygen-scavenging ability is critical for peroxyl radical scavenging [7]. The singlet oxygen absorbance capacity (SOAC) assay [8] was developed to measure the singlet oxygen. Research on the effects of foods and compounds on singlet oxygen is ongoing. The Antioxidant and Functional Research Group [9], established in Japan to promote research on antioxidant and functional properties of foods, named the general antioxidant capacity of foods antioxidant unit and defined it as the sum of ORAC and SOAC measurements.

The analysis of antioxidant capacity is time consuming and expensive. To screen compounds from candidates, the development of a simple and rapid method for measuring the antioxidant capacity of compounds is critical. Machine learning was widely used in various fields, such as industry, medicine, and engineering, for efficient research, and diagnosis [10,11]. In the chemical field, the use of machine learning is promoted in domains such as toxicity determination [12], retrosynthesis [13], and prediction of the antioxidant capacity of polyphenols [14]. Machine learning can efficiently evaluate physical properties and provide details for searching the structure of new substances. In chemical studies, preparing a large amount of data for training is difficult, and machine learning should be performed with a small amount of data. Therefore, a method to obtain accurate predictions from a small amount of data is necessary.

In this study, a regression model that predicts singlet-oxygen-scavenging capacity as an objective variable was constructed by using the molecular structure as an explanatory variable. The numerical values obtained from computational chemistry and Morgan fingerprinting [15] were used as the input data. To obtain the interpretability of the machine learning model, regression coefficients were obtained from the linear model, and the feature importance was obtained from the prediction model based on the decision tree [16]. To verify the interpretability of the model, we attempted to explain the reaction mechanism [17] of singlet oxygen scavenging from the feature importance.

## 2. Experiment

### 2.1. Preparing Dataset

The singlet-oxygen-scavenging capacities of 74 compounds were used in the dataset. The SOAC values of compounds were obtained from previous studies [8]. The SOAC values can be calculated as the ratio of the quenching rate constants of the antioxidants and α-tocopherol.

**Figure 1:**
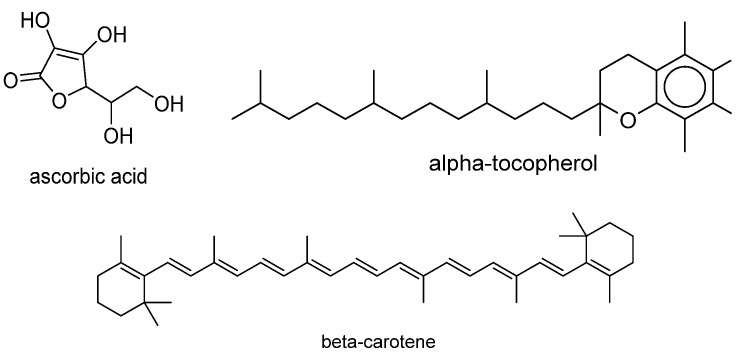
Examples of compounds in dataset.

SOAC values for the compounds whose values are not available in literature but whose singlet-oxygen-scavenging rates are known were determined by calculating the ratio of the quenching rate constants of the antioxidant and α-tocopherol. The natural logarithm of the rate ratio was used as the objective variable. Examples of compounds in the dataset are displayed in Figure 1. The names of all compounds in the dataset and their singlet-oxygen-scavenging capacities are listed in Table S1 of the Supporting Information.

The compounds data were obtained from PubChem as isomeric SMILES (Simplified Molecular Input Line Entry System) [18]. The most stable structure was determined as the initial structure for each sample by exploring the coordination from the molecular force field calculation using Balloon [19]. Then, the most stable structure was used as the initial structure. From the obtained structures, molecular descriptors and Morgan fingerprints were output using RDkit [20] and used as explanatory variables for the dataset. Using the most stable structure, PM7 [21] calculations were performed using MOPAC (Molecular Orbital PACkage) [21]. The PM7 method, which is a semiempirical method, was applied to optimize the structure under the assumption of vacuum conditions. We obtained HOMO (Highest Occupied Molecular Orbital), LUMO (Lowest Unoccupied Molecular Orbital), formation heat, and dipole moment values for each compound via PM7. Because PM6 is as accurate as density functional theory (DFT) calculations [22,23], we assumed that the PM7 calculations exhibit identical acceptable errors as DFT calculations under the B3LYP/6-31G* condition.

Feature selection was performed using more than 100 descriptors generated through RDkit and PM7. Firstly, descriptors with zero variance or duplicate content were deleted. Secondly, the correlation coefficients between explanatory variables were calculated. For the two descriptors with the highest absolute values of correlation coefficients, one of the descriptors was deleted. Descriptors with high correlation coefficients with many variables were omitted from the dataset because one indicator could substitute for many descriptors. Therefore, the number of descriptors used in the dataset was 61. The list of descriptors is provided in the Supporting Information.

### 2.2. Machine Learning Model

In this study, XGBoost [24,25], LightGBM [12,26], CatBoost [27], random forest [28], AdaBoost [29], LASSO regression [30], and the deep neural network (DNN) [31] were used to construct machine learning models. Additionally, we used Scikit-learn [32], TensorFlow [33], and Keras [34] to construct the machine learning model. The hyperparameters were set as described in the Supporting Information. The compound data were randomly categorized into 66 training data and 8 test data by setting the random state values to 0, 10, and 100. The same machine learning model was trained on molecular descriptors and Morgan fingerprints separately to evaluate and compare prediction performance. In this study, we compared the prediction accuracy using the coefficient of determination *R*^2^ and root mean squared error (RMSE) and used the leave-one-out cross-validation (LOOCV) method to ensure sufficient level of learning. In LOOCV, one sample from the original set was removed and used as the validation sample, and the rest was used for training. By changing a validation sample, *n* training datasets were created when the original training dataset had *n* samples. The output value by LOOCV (RMSE_LOO_) is the average value of the RMSE obtained by evaluating the machine learning model with each validation sample.

### 2.3. Feature Importance

The contribution of each feature to the prediction was analyzed by using the absolute values of coefficients obtained by LASSO regression and feature importance obtained by random forest, XGBoost, LightGBM, CatBoost, and AdaBoost. To analyze feature importance, we created a feature ranking for each machine learning model [16]. We focused on prediction models with *R*^2^ ≥ 0 and analyzed the feature importance. For each model, the top-10 important features were assigned a score in the order of importance: 10, 9, 8, 7, 6, 5, 4, 3, 2, and 1. By summing the scores of the prediction models and comparing them, we identified the important variables in the machine learning model for antioxidant capacity.

## 3. Results

### 3.1. Prediction

**Figure 2:**
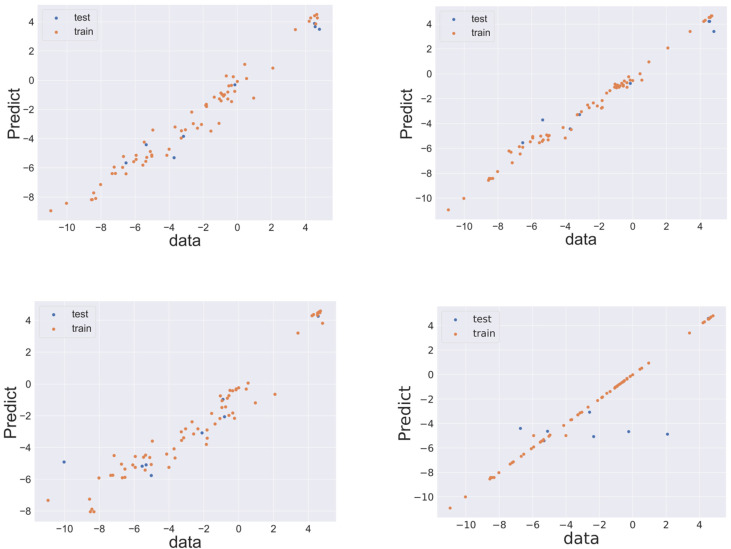
Relationship between objective feature values in the dataset and the predicted values. **Top row**: descriptors/random forest (**left**), and Morgan fingerprint/AdaBoost (**right**); **Bottom row**: Morgan fingerprint/random forest (**left**), and Morgan fingerprint/XGBoost (**right**). In all panels, the blue and orange markers indicate test and training data, respectively.

The performance of machine learning was estimated using LOOCV. To evaluate the prediction performance of each algorithm, RMSE_LOO_ was calculated. Table 1 lists the values of the evaluation indices for prediction accuracy when predicting the test data for each model. Comparison of the datasets revealed that superior predictive performance was achieved when molecular descriptors were used as explanatory variables. In AdaBoost and LASSO regression, superior performance was obtained when Morgan fingerprints were used as the explanatory variable. The optimal performance was obtained when random forest was combined with molecular descriptors as explanatory variables. Random forest exhibited robustness of the prediction model as *R*^2^ did not become negative. When the Morgan fingerprint was used as an explanatory variable, the performance of AdaBoost was the highest, followed by random forest and XGBoost. In the case of AdaBoost, the coefficient of determination was sometimes negative, whereas *R*^2^ was greater than zero in the case of XGBoost or random forest. XGBoost and random forest exhibited prediction accuracy comparable to AdaBoost and robustness superior to that of AdaBoost. Figure 2 displays the relationship between the values of the target variables (literature values) and the predicted values of the datasets for the random forest model trained with molecular descriptors and the AdaBoost, random forest, and XGBoost models trained by Morgan fingerprints. The relationship between literature values and predicted values in other cases is shown in the Supporting Information.

### 3.2. Importance Analysis

We focused on the sum of the importance scores of all models to understand the relationship between the structure and singlet-oxygen-scavenging capacity. The top seven features and their scores in the order of summation are listed in Table 2. The ranking of the importance obtained from each model is displayed in the Supporting Information. From the molecular descriptor dataset, HOMO, HOMO–LUMO gap, SlogP_VSA2, SlogP_VSA4, SlogP_VSA6, and PEOE_VSA7 values were obtained. SlogP_VSA2, SlogP_VSA4, SlogP_VSA6 and PEOE_VSA7 are molecular descriptors representing atomic contributions to logP and atomic partial charge [35]. They are based on van der Waals surface area. BalabanJ is a topological index based on the sum of distance of each bond in a certain molecule [36].

**Figure 3:**
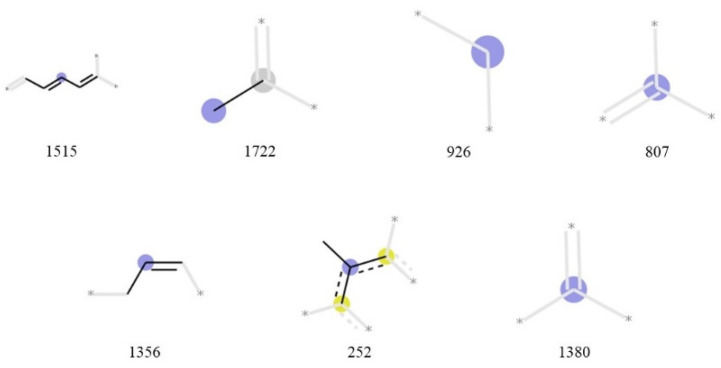
Important Morgan fingerprint bits. Bit 1515 suggests conjugate systems in a molecule. Bit 1722 represents a methyl group. Bits 926, 807, and 1380 represent carbon atoms. Bit 1356 suggests an allyl group or an unsaturated aliphatic carbon ring. Bit 252 represents an aromatic ring that has a methyl group.

Figure 3 displays the partial structure of each bit considered important in the machine learning using the Morgan fingerprint. Bits 1515 and 252 indicate that the presence or absence of a conjugated system considerably affects the singlet-oxygen-scavenging ability. Bit 1722 represents a methyl group attached to an aliphatic carbon atom, and bit 926 represents a carbon chain or a carbon atom with two C-H bonds. Bits 807, 1356, and 1380 represent carbon atoms with double bonds and single bonds. Bits 807 and 1380 exhibit no information regarding bond destination of each carbon atom.

## 4. Discussion

### 4.1. Prediction Accuracy

An excellent prediction performance was obtained by using the proposed machine learning method. Predictions without computational chemistry and using computational chemistry were both possible, which indicated that machine learning can be useful for understanding the antioxidant capacity. Machine learning exhibits considerable flexibility to provide prediction accuracy according to the user’s objectives by using various explanatory variables, which is critical for practical applications. Each bit of the Morgan fingerprint is represented by 0 and 1, respectively. Furthermore, the substructure from the central atom to at most two bonds ahead is used as the feature value. Therefore, the length of the conjugated system is not reflected, and counting the number of locations that have the same substructure is not feasible. Therefore, the prediction results using the Morgan fingerprint were less accurate than those using molecular descriptors. LASSO regression was an exception to this trend, but more variables can improve prediction accuracy. DNNs did not perform well on either dataset in this study. Thus, the simple DNN was not suitable for predicting the antioxidant capacity using a small amount of data.

Comparing the RMSE during cross-validation with the RMSE when evaluated on the test data, we determined that the machine learning models did not overfit. We also revealed that the Morgan fingerprint tends to overfit our models. Whether or not overfitting occurs depends on the size of the data, especially the number of features.

### 4.2. Importance Analysis

The results of the Morgan fingerprint revealed that conjugated systems and carbon atoms with double and single bonds are of particular importance. As the conjugated chain became longer, the absorption due to the π–π* transition shifted to the longer wavelength side [37]. This result indicated that the HOMO–LUMO gap became narrower, which is consistent with the high importance of the HOMO and HOMO–LUMO gaps in molecular descriptor datasets.

The electronic energy transfer (EET) [17] is expressed as Equation (1), in which the quencher transitions to the triplet state upon elimination of singlet oxygen and exhibits a rate constant close to the diffusion-rate-limiting rate of the quenching mechanism of singlet oxygen.

O_2_(^1^Δ_γ_) + Σ_0_→O_2_(^3^Σ_g_^−^) + T_1_ (1)

**Scheme 1:**
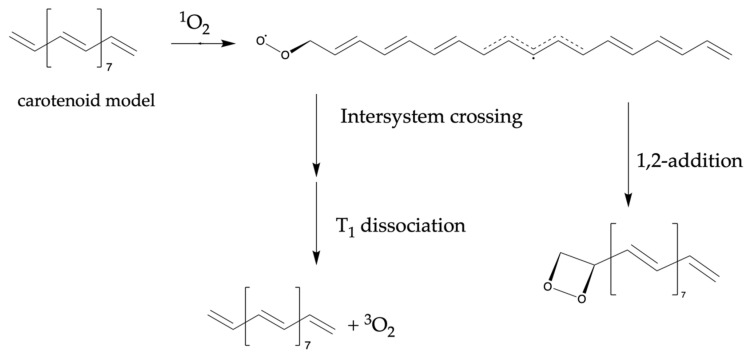
Competitive reactions in electron energy transfer (EET) mechanisms considered as chemical quenching.

In this scheme, the narrow HOMO–LUMO gap implied the ease of energy exchange between the quencher and singlet oxygen. In the EET mechanism, an encounter complex is formed by singlet oxygen and an antioxidant in the singlet state, and energy transfer is proposed to occur through the term crossing of the complex [38]. The larger HOMO value suggested that the antioxidant is more likely to approach the singlet oxygen, which is an electrophilic agent, thus promoting energy transfer. The reaction mechanism reported as a competitive reaction in this scheme is displayed in Scheme 1 [39]. The quencher and oxygen reacted to form a complex that underwent radicalization. Subsequently, oxygen chemical quenching occurred, or peroxides and carbonyl compounds were formed. Therefore, HOMO is expected to be used as an indicator of nucleophilicity from antioxidants to oxygen during chemical quenching.

In the quenching of singlet oxygen by phenols, two types of physical quenching reactions are known, namely, electrons are transferred between the aromatic ring and oxygen in the transition state but no oxygen is consumed, and chemical quenching, in which peroxides are formed [40]. The EET mechanism was consistent with machine learning inference because the reaction rate of the EET mechanism is close to the diffusion rate.

As mentioned earlier, singlet-oxygen-scavenging activity is correlated with the length of the conjugated chain, the length of the carbon chain, and the absorption wavelength of the ground state. The Morgan fingerprint bit displayed in Figure 3 is a critical indicator that can be explained using the EET mechanism as well as the HOMO and HOMO–LUMO gap because it can represent the structure of the conjugated system or a part of it. Because fingerprints can be used for machine learning, the substructure of the compound could be used as an alternative indicator to the HOMO.

Because SlogP_VSA2, SlogP_VSA4, SlogP_VSA6, and PEOE_VSA7 are critical when molecular descriptors are used as a dataset, atomic distribution for solubility, and partial charge were important in predicting antioxidant capacity. Reorganizing the dataset and examining new descriptors of electron density or polarity can improve the prediction performance and reveal electronic effects that are critical for studying antioxidants.

Analyzing the behavior of machine learning models by feature importance can explain prediction accuracy. Although feature importance is an ineffective measure for explaining causality, we interpreted it chemically by comparing it to previously known information. The process of testing hypotheses formulated by machine learning with computational chemistry and experiments is useful not only for efficiently evaluating properties that previously relied solely on experiments, such as antioxidant capacity, but also for verifying the validity of the evaluation.

## 5. Conclusions

A critical challenge in applying machine learning in chemistry and life science is that the prediction process remains unclear and the amount of data to be collected is small. In this study, we developed a prediction model that is easily interpretable by chemists and requires only a small amount of data. The proposed machine learning model can predict singlet-oxygen-scavenging activity of compounds, which is critical in food science. Molecular descriptors and Morgan fingerprints were used to validate the simple antioxidant capacity by the proposed method, and the importance of the features and interpreted the behavior of the machine learning model were examined chemically. Thus, the evaluation of antioxidant capacity was simplified and did not require time-consuming experiments. The prediction mechanism was also explained.

## Figures and Tables

**Table 1:** Regression metrics values of machine learning using estimator candidates.

Model	Dataset	Random_State	RMSE	*R* ^2^	RMSE_LOO_
XGBoost	descriptors	0	1.2913	0.9147	1.7566
		10	1.6867	0.8279	
		100	2.0362	0.6968	
	fingerprint	0	1.0857	0.9397	1.7857
		10	2.7150	0.5542	
		100	3.1873	0.2325	
LightGBM	descriptors	0	2.4106	0.7028	1.8723
		10	1.8021	0.7546	
		100	1.8021	0.7546	
	fingerprint	0	3.7413	0.2841	2.4055
		10	2.9596	0.4702	
		100	3.4717	0.0894	
CatBoost	descriptors	0	2.0570	0.7836	1.7375
		10	2.1533	0.7196	
		100	2.4917	0.5309	
	fingerprint	0	2.1026	0.7739	2.5477
		10	2.3711	0.6600	
		100	3.6750	−0.0200	
Randomforest	descriptors	0	1.0123	0.9476	1.5731
		10	1.9342	0.7737	
		100	1.9166	0.7225	
	fingerprint	0	1.3950	0.9005	1.7613
		10	1.2796	0.9163	
		100	2.0136	0.7548	
AdaBoost	descriptors	0	0.8141	0.9661	1.7006
		10	2.1277	0.7262	
		100	1.9945	0.6995	
	fingerprint	0	0.9573	0.9531	1.6017
		10	1.8246	0.7986	
		100	3.8793	−0.1370	
LASSO	descriptors	0	2.1668	0.7599	2.3314
		10	2.4023	0.6510	
		100	5.5896	−1.3605	
	fingerprint	0	1.7224	0.8483	1.9853
		10	3.0045	0.4540	
		100	4.0502	−0.2393	
DNN	descriptors	0	2.0145	0.7924	2.9865
		10	2.6807	0.5653	
		100	3.6878	−0.0275	
	fingerprint	0	1.8309	0.8286	3.3584
		10	3.3159	0.3350	
		100	3.4791	0.0855	

**Table 2:** Feature importance points of features with a large contribution for prediction.

Descriptor	Score	Fingerprint	Score
HOMO	175.5	1515	148.5
HOMO–LUMO gap	109	1722	118.5
SlogP_VSA4	84.5	926	93.5
SlogP_VSA2	72.5	807	87.5
SlogP_VSA6	72	1356	80
PEOE_VSA7	62	252	58.5
BalabanJ	44	1380	35.5

HOMO, Highest Occupied Molecular Orbital; LUMO, Lowest Unoccupied Molecular Orbital; VSA, Van der Waals Surface Area; PEOE, Partial Equation of Orbital Electronegativity.

## Data Availability

All of the data is contained within the article and the Supplementary Materials.

## Supplementary Materials

The following are available online at https://www.mdpi.com/article/10.3390/antiox10111751/s1, Table S1: The Dataset; Figures S1–S5: Relationship between the objective variable of the data set and its predictive values; Table S2: Feature importance in ensemble learning and coefficient of linear regression.
