# Supplementary material for: Prediction and Chemical Interpretation of Singlet-Oxygen-Scavenging Activity of Small Molecule Compounds by Using Machine Learning
Source: Antioxidants (Basel). 2021 Nov 1;10(11):1751. doi: 10.3390/antiox10111751 (PMC8614782; doi:10.3390/antiox10111751)
Supplement: Supplementary file 1 [file antioxidants-10-01751-s001.zip › antioxidants-1436592-supplementary.pdf]

Supporting Information

Prediction and Chemical Interpretation of Singlet Oxygen Scavenging Activity of Small Molecule Compounds Using Machine Learning

Department of Chemistry and Bioscience, Graduate School of Engineering Science, Yokohama National University

○Taiki FUJIMOTO, Hiroaki GOTOH*

1. data set

The compound names and natural logarithm of singlet oxygen scavenging capacity for the data set used in this study are shown in Table S1. Singlet-oxygen-scavenging capacity values of 74 compounds are obtained from papers.[1,2] LN(SOAC) means natural logarithm singlet-oxygen-scavenging capacity values.

Table S1 The Dataset

Name	LN(SOAC)
1_4_benzoquinone	-1.0788
2_3_dihydro_22467_pentamethylbenzofuran_5_ol	0.5390
2_3_dimethoxy_5_methyl_1_4_benzenediol	-0.8145
2_3_dimethyl_1_4_benzenediol	-0.9045
2_4_6_tritertbutylphenol	-5.4638
2_4_dimethylphenol	-1.5523
2_4_dimethyl_2_pentene	-5.5445
2_5_dimethyl_1_4_benzenediol	-0.7520
2_6_bis1_1_dimethylethyl_4_phenylphenol	-6.6816
2_6_dimethylphenol	-10.9449
2_6_dimethyl_1_4_benzenediol	-1.3581
2_methyl_1_4_benzenediol	-0.5596
2_methyl_2_pentene	-5.3738
2_naphthol	-7.3456
3_4_dimethylphenol	-7.1562
3_hydroxyflavone	-8.0258
4_aminophenol	-1.8563
4_methylcatechol	-3.0491
4_methyl_1_3_pentadiene	-6.5331
acetic_acid	-10.0261
adrenaline	-5.1073
all_e_retinal	-3.2968
alpha_carotene	4.5261
alpha_cehc	-0.1542
alpha_tocopherol	0
astaxanthin	4.6913
beta_carotene	4.5623
beta_cryptoxanthin	4.2136
beta_tocopherol	-0.1439
bht	-0.3403
biliverdin	0.4308
capsanthin	4.5981
catechol	-5.3154
cholesterol	-2.3418
caffeic_acid	-5.0222
dabco	0.9555
delta_tocopherol	-0.9676
dihydroxylycopene	3.4012
dopamine	-5.9402
dpbf	2.0794
epicatechin	-2.5876
epicatechin_gallate	-3.2834
epigallocatechin	-2.1037
epigallocatechin_gallate	-3.1606
etretinate	-0.9555
e_2_pentene	-8.5637
e_3_methyl_2_pentene	-4.0073
e_4_octene	-8.5172
ferulic_acid	-6.0836
gamma_tocopherol	-0.3481
histidine	-3.7114
hydroquinone	-5.3496
isoeugenol	-3.6574
lutein	4.3014
lycopene	4.8122
methyl_gallate	-6.7338
nn_diphenyl_p_phenylenediamine	-0.2412
resorcinol	-5.0192
retinyl_acetate	-4.9619
tocol	-1.8202
trimethyl_1_4_benzenediol	-0.5596
trolox	-1.0527
vitamine_c	-4.1352
zeaxanthin	4.5304
z_3_methyl_2_pentene	-5.9384
z_4_methyl_4_octene	-6.5331
z_4_octene	-8.3124
z_9_tricosene	-8.4300
canthaxanthin	4.6600
quercetin	-7.2300
6_hydroxy_22578_pentamethylchroman	-0.5030
2_2_methylenebis4_methyl_6_tert_butylphenol	-2.6800
2_5_di_tert_butylhydroquinone	-1.8100
ubiquinol10	-0.6520

2. descriptor

61 molecular descriptors used in this study are shown below. Structures of 74 compounds were obtained as canonical SMILES from PubChem.[3] Molecular 61 descriptors and Morgan fingerprint are obtained from SMILES by using RDkit.[4] Then, heat of formation, HOMO, LUMO, dipole moment 74 compounds are calculated with PM7, one of the semi-empirical molecular orbital methods by using MOPAC.[5]

Molecular descriptors generated by RDkit
MaxEStateIndex, MinEStateIndex, qed, MaxPartialCharge, MinPartialCharge, FpDensityMorgan1, FpDensityMorgan3, BalabanJ, BertzCT, HallKierAlpha, Ipc, Kappa2, PEOE_VSA10, PEOE_VSA11, PEOE_VSA12, PEOE_VSA13, PEOE_VSA14, PEOE_VSA2, PEOE_VSA3, PEOE_VSA7, PEOE_VSA8, PEOE_VSA9, SMR_VSA10, SMR_VSA3, SMR_VSA4, SMR_VSA7, SlogP_VSA1, SlogP_VSA10, SlogP_VSA11, SlogP_VSA2, SlogP_VSA3, SlogP_VSA4, SlogP_VSA5, SlogP_VSA6, SlogP_VSA8, TPSA, EState_VSA1, EState_VSA10, EState_VSA2, EState_VSA3, EState_VSA4, EState_VSA5, EState_VSA6, EState_VSA7, EState_VSA8, EState_VSA9, VSA_EState1, VSA_EState2, VSA_EState4, VSA_EState5, VSA_EState6, VSA_EState8, VSA_EState9, FractionCSP3, MolLogP, MolMR

Descriptors obtained by PM7 calculation
heat of formation, HOMO, LUMO, HOMO-LUMO gap, dipole moment

3. hyperparameter

The following is a list of parameters that were set when the prediction model was developed.
CatBoost
learning_rate = 0.024, n_estimators = 1000, loss_function = 'RMSE', depth = 7, early_stopping_rounds = 10

LightGBM, XGBoost
num_leaves = 100, n_estimators = 1000, max_depth = 7, learning_rate = 0.02

random forest
max_depth = 7, n_esimators = 500

AdaBoost
base_estimator = DecisionTreeRegressor(max_depth = 7), learning_rate = 0.02, n_estimators = 1000

Lasso regression

With molecular descriptors as explanatory variables: alpha = 1, max_iter = 10000

Morgan fingerprint as explanatory variable: alpha = 0.01, max_iter = 5000

neural network

When molecular descriptors are used as explanatory variables

4 layers including input/output layers

Number of nodes in each layer: 61, 32, 16, 1

Loss function (loss): Mean squared error

Metrics: Mean absolute error

Activation function: constant for output layer, ReLu for others

Optimizer: Adam

EarlyStopping (monitor = 'loss', patience=10)

batch_size = 32, epochs = 500

When Morgan Fingerprint is as an explanatory variable

6 layers including input/output layers

Number of nodes in each layer: 2048, 1024, 512, 256, 128, 1

Loss function (loss): Mean squared error

Metrics: Mean absolute error

Activation function: constant for output layer, ReLu for others

Optimizer: Adam

EarlyStopping (monitor = 'loss', patience=10)

batch_size = 32, epochs = 500

4. Relationship between the objective variable of the data set and its predictive values

Figure S1-5 shows the relationship between the objective variables and their predictions for the dataset with random_state = 0 among the predictive models. The orange plots represent the training data, and the blue plots represent the test data. If the orange plots are lined up in a straight line, it means that the training is sufficient. If the orange plots are not lined up, it means that the training is insufficient, or an unsuitable algorithm is used.

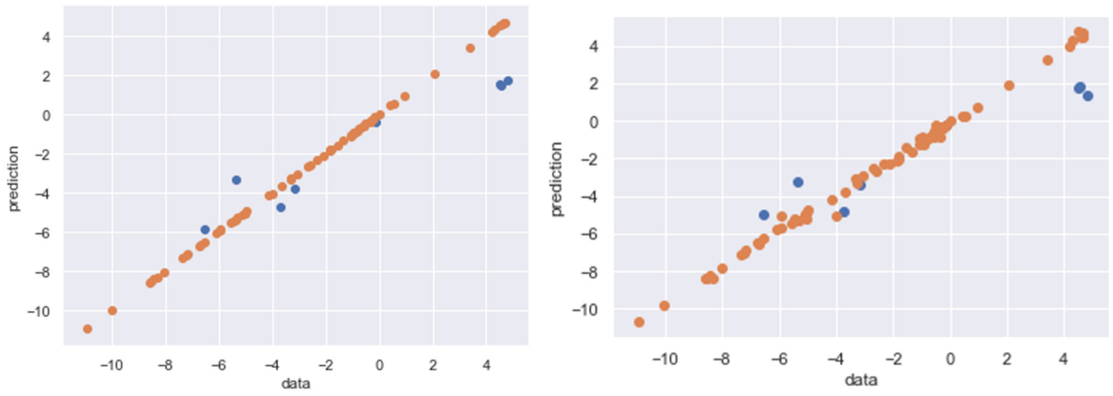

Figure S1 descriptors/CatBoost and Morgan Fingerprint/CatBoost

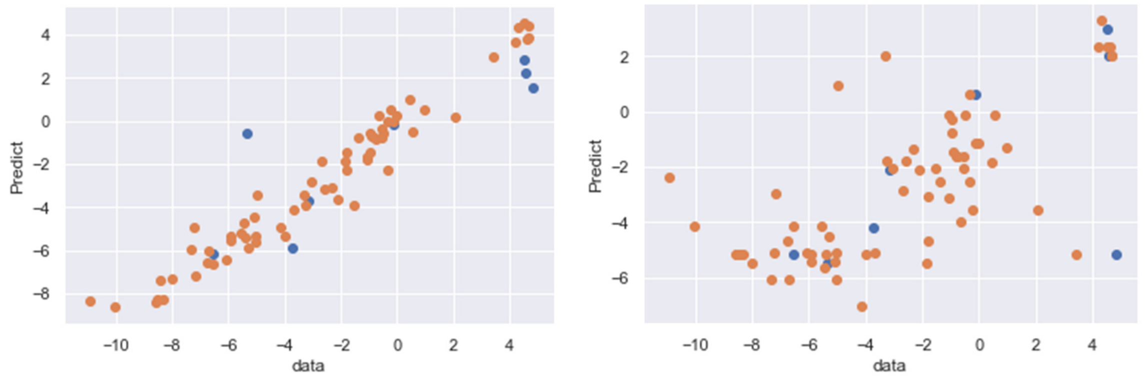

Figure S2 descriptors/LightGBM and Morgan Fingerprint/LightGBM

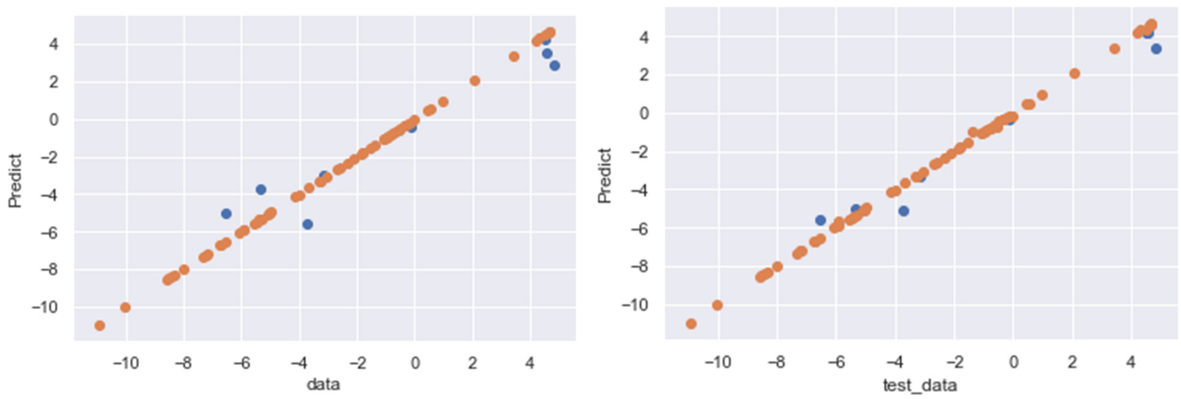

Figure S3 descriptors/XGBoost and descriptors/Adaboost

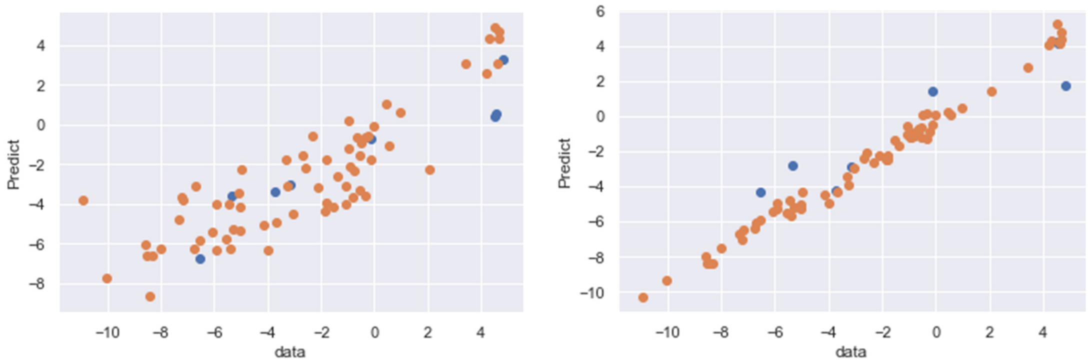

Figure S4 descriptors/Lasso and Morgan Fingerprint/Lasso

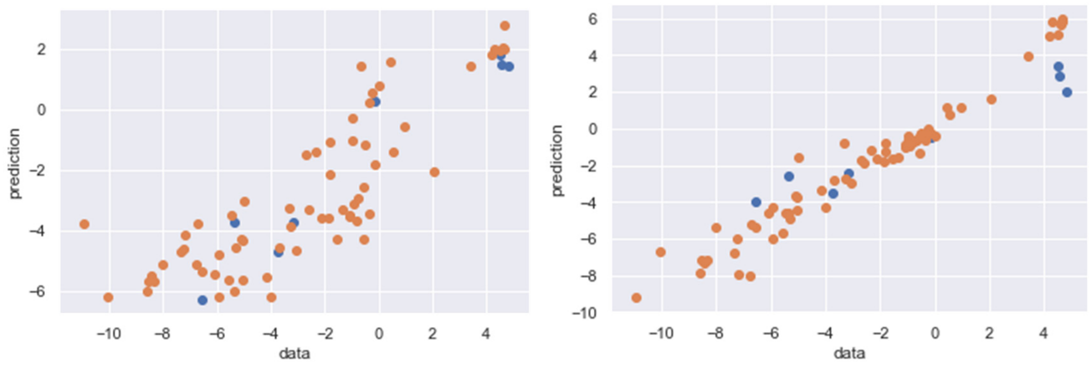

Figure S5 descriptors/Deep Neural Network and Morgan Fingerprint/Deep Neural Network

5. Importance ranking

The ranking of the importance obtained from each prediction model other than the multilayer neural network is shown as Table S2. From left to right, the importance is higher, and the number of points in the importance point is added to each feature, and Table 2 is created.

Table S2 Feature importance in ensemble learning and coefficient of linear regression

Model	dataset	random_state	importance point									
			10	9	8	7	6	5	4	3	2	1
CatBoost	descriptors	0	HOMO	LUMO-HOMO	SlogP_VSA4	SlogP_VSA6	MolMR	Estate_VSA2	VSA_EState8	Kappa2	BalabanJ	VSA_EState6
		10	HOMO	SMR_VSA7	SlogP_VSA6	PEOE_VSA7	LUMO-HOMO	VSA_EState6	SlogP_VSA4	MolMR	Ipc	PEOE_VSA10
		100	HOMO	SMR_VSA7	SlogP_VSA4	SlogP_VSA6	LUMO-HOMO	PEOE_VSA7	VSA_EState6	SlogP_VSA11	VSA_EState4	Ipc
	Morgan fp	0	1515	1722	1356	926	807	55	1380	1060	679	252
		10	1515	1722	432	807	294	1356	926	1742	173	1626
XGBoost	descriptors	0	HOMO	PEOE_VSA2	LUMO-HOMO	SMR_VSA7	Estate_VSA2	HallKierAlpha	SlogP_VSA1	VSA_EState8	SlogP_VSA2	SlogP_VSA3
		10	HOMO	SLogP_VSA1	VSA_EState1	SlogP_VSA2	SMR_VSA7	VSA_EState5	VSA_EState8	PEOE_VSA2	SlogP_VSA3	SlogP_VSA4
		100	HOMO	SlogP_VSA6	SlogP_VSA3	SlogP_VSA4	MinPartialCharge	PEOE_VSA2	SlogP_VSA8	VSA_EState8	SlogP_VSA2	VSA_EState4
	Morgan fp	0	1515	252	801	872	191	926	807	420	90	750
		10	1515	252	750	191	807	694	146	926	1356	801
		100	1515	252	1823	926	875	1057	420	801	1607	1750
LightGBM	descriptors	0	HOMO	BalabanJ	PEOE_VSA7	SlogP_VSA2	MinPartialCharge	FpDensityMorgan1	SlogP_VSA4	LUMO	SlogP_VSA6	Estate_VSA2
		10	HOMO	PEOE_VSA7	LUMO	BalabanJ	FpDensityMorgan3	qed	FpDensityMorgan1	VSA_EState6	Estate_VSA4	MinPartialCharge
		100	HOMO	PEOE_VSA7	LUMO	BalabanJ	FpDensityMorgan3	qed	FpDensityMorgan1	VSA_EState6	Estate_VSA4	MinPartialCharge
	Morgan fp	0	1722	926	1602	875	80	1607	1750	1873		
		10	1060	1722	926	1602	80	875	1750	1873	694	
		100	926	1722	1017	694	875	1873	1602	1750	80	
random forest	descriptors	0	HOMO	LUMO-HOMO	SlogP_VSA6	SlogP_VSA4	SlogP_VSA2	Estate_VSA5	MinPartialCharge	PEOE_VSA7	BalabanJ	LUMO
		10	HOMO	SlogP_VSA6	LUMO-HOMO	SlogP_VSA2	PEOE_VSA7	SlogP_VSA4	MinPartialCharge	Estate_VSA8	DIPOLE	BalabanJ
		100	HOMO	SlogP_VSA6	PEOE_VSA7	LUMO-HOMO	SlogP_VSA4	SlogP_VSA2	VSA_EState8	SMR_VSA7	MolMR	MinPartialCharge
	Morgan fp	0	1515	1722	252	1380	926	807	1356	1999	1692	1060
		10	1515	1722	1380	252	926	1356	807	1999	1692	1060
		100	1515	252	1722	807	1356	926	1380	1742	694	1039
Adaboost	descriptors	0	HOMO	LUMO-HOMO	BalabanJ	Estate_VSA5	SlogP_VSA6	SlogP_VSA4	MinPartialCharge	Estate_VSA4	SlogP_VSA2	SMR_VSA7
		10	HOMO	LUMO-HOMO	SlogP_VSA2	SlogP_VSA8	BalabanJ	SlogP_VSA4	MinPartialCharge	SMR_VSA7	Estate_VSA5	DIPOLE
		100	HOMO	SlogP_VSA6	LUMO-HOMO	PEOE_VSA7	Estate_VSA5	SlogP_VSA4	VSA_EState6	SlogP_VSA3	BalabanJ	SlogP_VSA2
	Morgan fp	0	1515	1722	926	807	1380	1999	1692	1356	508	252
		10	1515	1722	807	1356	1999	1039	926	252	1692	1380
Lasso	descriptors	0	LUMO-HOMO	HOMO	SlogP_VSA2	SlogP_VSA4	VSA_EState5	FpDensityMorgan3	SlogP_VSA5	PEOE_VSA8	SMR_VSA3	PEOE_VSA14
		10	LUMO-HOMO	SlogP_VSA2	HOMO	formation_HEAT	SlogP_VSA4	PEOE_VSA13	VSA_EState5	MinPartialCharge	DIPOLE	VSA_EState4
	Morgan fp	0	1356	1515	1692	503	807	508	872	1722	420	203
		10	1515	1356	1039	807	503	1804	1722	801	4	784

6. Reference

1. Mukai, K. Antioxidant Activity of Foods: Development of Singlet Oxygen Absorption Capacity (SOAC) Assay Method. *J. Nutr. Sci. Vitaminol. (Tokyo).* **2019**, *65*, 285–302, doi:10.3177/jnsv.65.285.
2. Wilkinson, F.; Helman, W.P.; Ross, A.B. Rate Constants for the Decay and Reactions of the Lowest Electronically Excited Singlet State of Molecular Oxygen in Solution. An Expanded and Revised Compilation. *J. Phys. Chem. Ref. Data* **1995**, *24*, 663–677, doi:10.1063/1.555965.
3. Kim, S.; Chen, J.; Cheng, T.; Gindulyte, A.; He, J.; He, S.; Li, Q.; Shoemaker, B.A.; Thiessen, P.A.; Yu, B.; et al. PubChem in 2021: New data content and improved web interfaces. *Nucleic Acids Res.* **2021**, *49*, D1388–D1395, doi:10.1093/nar/gkaa971.
4. RDkit Available online: https://www.rdkit.org/.
5. Stewart, J.J.P. Optimization of parameters for semiempirical methods VI: More modifications to the NDDO approximations and re-optimization of parameters. *J. Mol. Model.* **2013**, *19*, 1–32, doi:10.1007/s00894-012-1667-x.
